# The Reach of Depression Screening Preceding Treatment: Are There Patterns of Patients' Self-Selection?

**DOI:** 10.1155/2012/148145

**Published:** 2012-11-06

**Authors:** Dea Ajduković, Mirjana Pibernik-Okanović, Mario Šekerija, Norbert Hermanns

**Affiliations:** ^1^Unit for Psychological Medicine, Vuk Vrhovac University Clinic, Merkur Teaching Hospital, Zajčeva 19, 10000 Zagreb, Croatia; ^2^Service for the Epidemiology of Non-Communicable Diseases, Croatian National Institute of Public Health, Rockefellerova 7, 10000 Zagreb, Croatia; ^3^Forschungsinstitut Diabetes-Akademie Bad Mergentheim (FIDAM GmbH), Diabetes Zentrum Mergentheim, Johann-Hammer-Straße 24, 97980 Bad Mergentheim, Germany

## Abstract

This study evaluated the reach of depression screening followed by treatment programs for subsyndromal depression and explored demographic and clinical characteristics of patients who were reached versus those who were not. A two-item Patient Health Questionnaire-Depression was sent to 4196 type 2 diabetic patients. Positively screened patients were interviewed to assess the severity of depression, and those with subclinical symptoms were invited to treatment groups. The reach of screening procedure was evaluated by the total response rate, proportion of positive depression screenings, and proportion of eligible patients entering treatment programs. Predictors of responsiveness to screening and of participation in treatment were determined using logistic regression. Of the 34% of patients who returned the questionnaire (*n* = 1442), 40% reported depressive symptoms and a need for professional help (*n* = 581). Age (OR = 1.06, 95% CI = 1.05–1.08), BMI (OR = 1.02, 95% CI = 1.00–1.04), HbA1C (OR = .92, 95% CI = .86–.99), and LDL-cholesterol (OR = .90, 95% CI = .81–1.00) correlated with response to screening. Willingness to accept treatment was predicted by professional status (OR = 3.24, 95% CI = 1.53–6.87), education (OR = 1.21, 95% CI = 1.05–1.38), and BMI (OR = .91, 95% CI = .85–.98). Older patients with better diabetes control were more likely to be reached by postal screening for depressive symptoms. Professionally inactive, better-educated persons and those with lower BMI were more likely to participate in the intervention for subsyndromal depression.

## 1. Introduction

Elevated depressive symptoms are common in diabetic patients, implying impaired quality of life [1] as well as difficulties in self-managing diabetes [2] and achieving desirable metabolic control [3]. Depression increases the risk for diabetic complications and mortality, not only in patients with severe forms of depression but also in those with mild, that is, subclinical, depressive symptoms [4–6].

Despite the clear evidence that the interaction between depression and diabetes is associated with adverse health outcomes, depression remains unrecognized in approximately half of diabetic patients and is consequently not treated properly [7, 8]. International guidelines currently advocate regular assessment of patients' well-being [9, 10] aimed at improving rates of recognition of emotional problems in people suffering from diabetes. These guidelines stress the importance of “incorporating psychological assessment and treatment into routine care rather than waiting for identification of a specific problem or deterioration in psychological status” [9]. The IDF guideline for type 2 diabetes mellitus advises to “assess well being and psychological status periodically, by questioning or validated measures.” The relevance of screening questionnaires to improve quality of care for depression has also been supported by the UK National Institute for Health and Clinical Excellence [11].

Many instruments assessing depressive mood have demonstrated sufficient sensitivity and specificity for detecting depressive symptoms in patients with diabetes [12, 13]. For screening purposes, short instruments focused on core depressive symptoms, such as the PHQ-2 [14], have been shown to be as useful as those containing a greater number of items, such as the CES-D or PHQ-9 [15, 16]. However, screening itself has not been shown to have an impact either on the recognition of depression or on its management and outcomes [17, 18]. In a recent RCT by Pouwer et al. [19], depression screening with written feedback to the patient and physician has not reduced depressive symptoms and has had a limited impact on the use of mental health service in comparison with care as usual. Similar findings were obtained in other patient populations [18, 20], suggesting that recommendations to adopt screening strategies are justified only if treatments are planned and provided.

Some studies in the field of diabetes have proven that combining assessment of emotional well-being with subsequent clinical interventions might be more promising in improving mood- and diabetes-related issues. A randomized controlled trial by Pouwer et al. [21] and a longitudinal study by Snoek et al. [22] have demonstrated that computerized assessment of psychological well-being followed by a discussion with a diabetes nurse specialist has improved psychological outcomes. In studies by Katon et al., positive effects of screening on depressive outcomes were observed when screening was embedded in a collaborative care intervention for depression [23, 24]. It can be concluded that the efficacy of screening depends on its integration into comprehensive treatment approaches including education for behavioral activation and self-management, followup, and, if necessary, referring patients to mental health services in order to intensify treatment.

In spite of the growing consensus on the value of early recognition of depressive symptoms in diabetic patients combined with appropriate interventions, there has been little published research on the reach of screening procedures in a real-world population of type 2 diabetic patients as well as on the proportion of patients who are willing to accept treatment for emotional difficulties. Also, little is known about whether diabetic persons who respond to depression screening and choose to enroll in depression treatment programs differ from those who do not in terms of demographic and disease-related characteristics.

This study was aimed to assess the reach of depression screening followed by treatment programs for subsyndromal depression and to explore demographic and clinical characteristics of patients who were reached versus those who were not. The study was a part of a three-arm randomized controlled trial comparing a six-week psychoeducational or physical exercise course and diabetes reeducation to address subclinical depression.

## 2. Materials and Methods

A cohort of 4196 type 2 diabetes patients was retrieved from a database of diabetic patients [25] based on the criteria of having type 2 diabetes, being between 18 and 65 years old, and having attended at least one diabetes specialist checkup in the previous year. Patients older than 65 years were assumed to be less likely to meet the inclusion criteria for the treatments following the screening procedure, particularly for physical exercise, thus making the recruitment process more complicated. In addition, literature data indicate that elderly diabetic patients with depressive symptoms have specific needs which have to be addressed in an adjusted way [26]. The study was carried out at the Vuk Vrhovac University Clinic, a referral centre for the treatment of diabetes, in Zagreb, Croatia. 

The patients were sent a letter explaining the importance of recognizing and treating depression in persons with diabetes and briefly informing them that a free-of-charge behavioral treatment program was available to patients who reported such difficulties [27]. However, specific descriptions of the available programs were not given at this point in the study.

The letter included a yes/no version of the two-item screening instrument for depression, the Patient Health Questionnaire (PHQ-2) [28], and an additional question inquiring into the patients' need to receive help in mood-related issues [29]. Adding the question about a need for help was shown to increase the specificity of the instrument [30].

The patients were provided with reply-paid envelopes and instructed to return the completed questionnaire regardless of their responses. They were also asked for permission to be contacted by phone if they indicated an interest in receiving help in mood-related issues.

Patients with positive screening results were telephoned to collect sociodemographic and personal data (professional, economic, and family status, and self-reported acute and chronic stress) and to assess the severity of depressive symptoms by administering a structured clinical interview (SCID-I). Persons who met criteria for major depressive disorder or dysthymia, patients with a history of serious psychiatric disorders (psychosis, bipolar affective disorder), and those who were receiving psychiatric treatment were not included in the treatment trial. Instead, they were advised to refer to their GP or psychiatrist, as they required more intensive treatment. Respondents were classified as having subclinical depressive symptoms if they did not meet diagnostic criteria for major depressive disorder or dysthymia. The rationale and the structure of the psychoeducational, physical exercise, and diabetes reeducation treatments were explained to the eligible patients, and they were asked for permission to be randomized to one of the groups. A general agreement to participate was considered the basis for randomization. Its results were presented to the patients at the first appointment, when their written consent was asked for.

Disease-related data including diabetes duration, BMI, HbA1C, total cholesterol, LDL, HDL, triglycerides, and albumin/creatinine ratio were extracted from the electronic files of the entire patient sample (*n* = 4196). Values obtained at the most recent medical checkup within the previous year were used for statistical analyses.

The reach of the screening procedure was evaluated based on the total response rate, the proportion of positive depression screenings, and the proportion of eligible patients who entered the treatment programs. Demographic and diabetes-related differences between patients who responded versus those who did not respond to screening, and between eligible patients who entered the treatment programs versus those who declined to participate were tested using one-way ANOVAs, *χ* ^2^ tests, and multivariate logistic regression analyses, and the effect sizes for comparisons of continuous variables were determined using Cohen's *d*.

## 3. Results

Of the 4196 PHQ-2 questionnaires sent to type 2 diabetic patients who were actively treated at the Vuk Vrhovac University Clinic for Diabetes, 1442 were returned, giving the response rate of 34%. Fifty-three letters were returned by the postal service because the recipient could not be reached, 19 patients denied having type 2 diabetes, and 16 patients were reported to have died.

Of the 34% of patients who returned the questionnaire (*n* = 1442), 40% reported at least one elevated depressive symptom and a need for professional help (*n* = 583). Approximately one-half of them (57%, *n* = 330) were considered eligible for treatment, while others were excluded based on the predefined criteria. Out of the eligible patients, 191 entered the treatment programs, 91 declined to do so, and 48 were unreachable after initial contact. Responsiveness to screening, reporting depressive symptoms, and expressing a need for help were comparable between female and male respondents (all *P*'s > 0.05).

**Figure 1:**
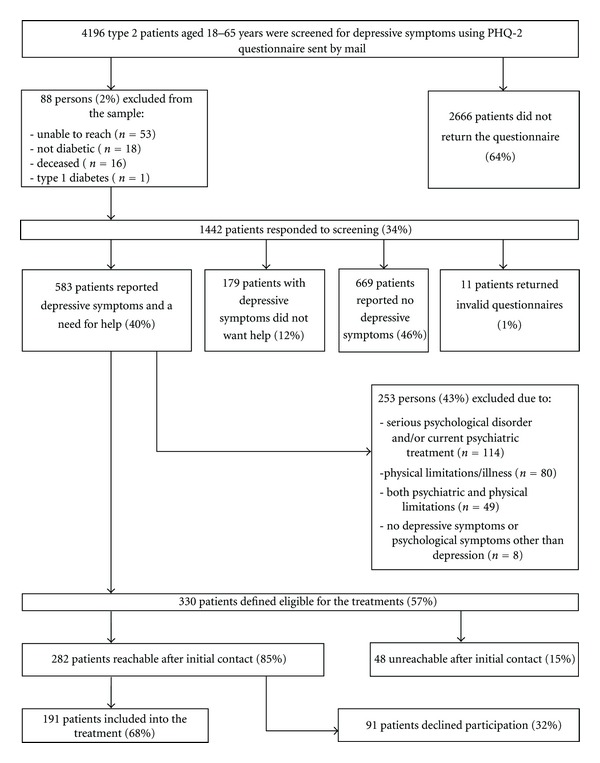
Flowchart of 4196 type 2 patients identified and recruited for trial participation.

A flow-chart of screening and recruiting patients for the treatment of subsyndromal depression is described in Figure 1.

A comparison between demographic and biochemical data of respondents and nonrespondents is given in Table 1. Univariate analyses revealed that respondents were older (58.5 ± 5.65 versus 55.4 ± 8.19 years, *P* < .001) and had lower HbA1C (7.1 ± 1.32 versus 7.2 ± 1.39%, *P* = .031) and LDL cholesterol (2.92 ± .976 versus 3.00 ± 1.046 mmol/L, *P* = .014) and higher HDL cholesterol (1.34 ± .327 versus 1.32 ± .322 mmol/L, *P* = .045). In the fully controlled multivariate model, age (OR = 1.06, 95%  CI = 1.05–1.08), BMI (OR = 1.02, 95%  CI = 1.00–1.04), HbA1C (OR = .92, 95%  CI = 0.86–.99), and LDL cholesterol (OR = .90, 95%  CI = 0.81–1.00) emerged as correlates of responding to the questionnaire. 

Demographic and diabetes-related data characterizing the subgroups of eligible patients who accepted the treatment versus those who did not accept it are given in Table 2. Treated patients differed from the patients who declined treatment at the point of signing consent with respect to BMI and triglycerides—both disease-related indicators were shown to be better in the treatment participants (*P* = 0.02 and *P* = 0.02, resp.). All other clinical characteristics including HbA1C, cholesterol, LDL, HDL, and albumin/creatinine ratio were comparable across the two groups (all *P*'s > 0.05). The proportion of professionally inactive patients was greater in the group of participating patients, while female gender reached borderline significance (*P* = 0.07). Other demographic variables including education, socioeconomic, and family status, as well as self-evaluations of acute and chronic psychological stress, were not shown to be associated with accepting or declining treatment.

Multivariate logistic regression analysis defining treatment participation as the dependent variable and BMI, HbA1C, LDL, HDL, triglycerides, gender, age, education, and socioeconomic, family, and professional status as predictors revealed professional inactivity (OR = 3.24, 95%  CI = 1.53–6.87), higher level of education (OR = 1.21, 95%  CI = 1.05–1.38), and lower BMI (OR = .91, 95%  CI = .85–.98) to be independent predictors of patients' readiness to be treated for subsyndromal depression.

## 4. Discussion

To the best of our knowledge, this is the first published study that investigated the diabetes-related characteristics associated with (non)responding to depression screening and willingness to participate in behavioral treatment of subsyndromal depression. A postal screening for depressive symptoms gave a 34% response rate, with 40% of the respondents reporting depressive mood and a need for help, and 12% expressing depressive symptoms but expressing no need for professional intervention in their mood-related difficulties. Whether the latter group was already treated, thus making any further treatment option unnecessary or other reasons governed their decision to decline help, remains a matter of speculation.

In several cross-sectional studies, postal screening was used to determine the prevalence of depressive symptoms in diabetic patients, yielding different response rates. In a study carried out in both metropolitan and rural areas in Australia [16], a response rate of 47% was obtained, while a study of older Australian adults reached 29% of the target sample [31]. A study by De Groot et al. [32] based in rural Appalachian counties gave a response rate of 46% (defined as answering to the letter of invitation), and an Irish study of patients with type 1 and type 2 diabetes had a response rate of 71% [33]. It could be speculated that the lower response rate obtained in our study was due to the fact that those who were not motivated to participate in the interventions were less likely to return the mailed questionnaire, despite the instructions asking to do so in any case.

Postal screening for depression by using the PHQ-2 has been shown to capture more positive screenings in medically ill older adults as compared with phone administration of the same questionnaire—15.1% versus 6.5% [34]. The authors suggest that postal screening by a combination of the PHQ-2, self-reported antidepressant use, and reported diagnosis of depression can capture the greatest number of persons with possible depression. The proportion of patients with elevated depressive symptoms reached by our screening procedure was 18% of the total number of patients who had been sent the questionnaire (*n* = 4196). Since the previously determined prevalence of depressive symptoms in type 2 diabetic patients treated at our clinic was 22% [35], it could be hypothesized that not all depressed type 2 patients were reached by the postal screening procedure. Among the 18% of patients who were contacted by telephone for a more detailed depression assessment, some were shown to have other possible psychological difficulties, such as anxiety, or insomnia, rather than elevated depressive symptoms. Therefore, the true proportion of depressed individuals who were not reached by the screening was probably underestimated.

Literature provides scarce evidence of demographic and clinical characteristics that may differentiate respondents and nonrespondents to depression screening. In this study, respondents were characterized by older age and relatively better indicators of diabetes management including glycaemic control and LDL and HDL cholesterol. Although these differences between the two groups could be interpreted in terms of statistical rather than clinical relevance, as shown by relatively small effect sizes, responsiveness to screening was independently predicted by diabetes-related indicators—BMI, HbA1C, HDL—and by age. In the studied cohort of type 2 diabetic patients, older individuals with better clinical status were more likely to return the questionnaire inquiring into their emotional state, regardless of whether they actually experienced depressive symptoms or not. Whether such self-selection may be analyzed in terms of differences in self-care and attitudes towards health-related issues remains to be clarified.

Of the respondents screened positively for depressive symptoms, 80 had been excluded from the interventions due to physical limitations or concomitant illnesses, 114 due to a previously diagnosed psychiatric disorder or current psychiatric treatment, 49 due to both psychiatric and physical limitations, and 8 due to no depressive symptoms detected by the structured clinical interview administered by phone or having psychological symptoms other than depression. These individuals were provided with information about treatment options or advised to start individual psychological and/or psychiatric treatment at our diabetes clinic.

About two-thirds of the eligible respondents (68%) were included into the structured treatments for subclinical depression—psychoeducation based on cognitive-behavioral principles, physical exercise, or diabetes reeducation. The remaining 91 declined participation, most frequently reporting competing priorities, such as professional and family obligations or lack of time as reasons for nonparticipation. The reach of behavioral interventions is increasingly considered important in determining the real impact of efforts to support patient well-being and diabetes self-care. Within the RE-AIM framework [36], reach is defined as the percentage of potential participants who are exposed to an intervention, and to their representativeness. The reach of 68% of eligible patients obtained in our study can be considered high, taking into account that it was achieved within the context of recruitment for an RCT, in which participants must be willing to take part in any of the treatment options to which they could be randomized. In comparison, the reach of a web-based cognitive behavioral therapy program to reduce symptoms of depression and diabetes-related distress [37] has been shown to be 47% of all interested patients.

A study by Glasgow et al. [38] demonstrated that giving eligible patients a chance to choose the treatment condition increased the participation rate in comparison with a randomized consent condition (48% versus 37%). 

Besides reach, representativeness of participating patients is considered relevant for translating RCT findings into regular clinical practice. In a study of internet-based weight loss programs [39] with a 30% participation rate, individuals with higher income, better education, and health literacy proficiency have been shown to be significantly more likely to participate. In a study by Toobert et al. [40], although the sample recruited for a lifestyle intervention in Latinas with diabetes was highly representative, participants and nonparticipants differed in the type of diabetes treatment. The observation that randomized patients may differ from those who were not randomized for intervention due to their unwillingness to participate has been confirmed in patients with irritable bowel syndrome as well [41], as patients with a higher intensity of abdominal pain and a longer disease history were shown to be more likely to participate in the intervention. In our study professionally inactive/retired, better educated, and less overweight persons were more likely to agree to participate in treatments for subsyndromal depression. On the other hand, the patients willing to participate did not differ from those refusing participation with respect to some demographic and psychological variables in which differences would be expected—such as the experience of acute or chronic stress. An assumption can be made that experience of stressful events is not necessarily associated with patients' needs to receive help but can act as both a motivator or an inhibitor of seeking help.

The obtained results allow a speculation that personal and disease-related indicators, but also specific life circumstances—such as being professionally inactive at the age in which active professional roles are expected—might contribute to patients' decision to accept the treatment. The effects of professional status may not be fully attributable to the issues of available time (since the interventions themselves offered flexibility in that regard) but also to the patients' psychological needs and attitudes towards health. Although differences between the two groups were small, their statistical significance justifies taking them into consideration in elaborating the applicability of final study results. An assumption can be made that devising interventions that would be better adjusted for professionally active patients, persons with a lower educational level, and those with higher body weight may be appropriate.

Another hypothetical way to increase patient participation in psychological treatments might be associated with a degree to which psychological issues are addressed in diabetes care in general. The more psychological topics are recognized and discussed within regular diabetes care, the greater readiness on patients' side to engage into treatments can be expected.

The presented data have some advantages and limitations. The main strength of this study is that it was carried out in a large cohort of type 2 diabetic patients. This allows a relatively broad generalizability of the findings on the characteristics associated with patients' willingness to respond to depression screening. However, the differences between persons who did and did not choose to participate in the treatment programs are small in magnitude and may be significantly influenced by the specifics of the design of the randomized clinical trial or of the studied subpopulation of diabetic persons. Therefore, further studies are needed to establish whether personal and disease-related characteristics predict the likelihood of participating in behavioral treatment for mood difficulties across different contexts and populations.

## 5. Conclusion

The processes of screening for depressive symptoms and recruiting type 2 diabetes patients for treatment of subsyndromal depression seem to be characterized by differences in demographic and clinical features of patients who were reached versus those who were not. The findings may be taken into consideration while evaluating the generalizability of trial results.

## Figures and Tables

**Table 1:** Demographic and clinical variables in patients who responded and did not respond to depression screening.

	Univariate analyses				Multivariate model	
	Responders	Nonresponders			Responders = reference	
	Mean ± SD (%)	Mean ± SD (%)	*P*	Cohen's *d *	OR (95% CI)	*P*
Gender						
(female = referent)	(44.8)	(43.2)	.323	N/A	1.04 (.90–1.21)	.608
Age	58.2 ± 5.92	55.6 ± 8.00	<.001	.369	1.04 (1.03–1.05)	<.001
BMI	30.0 ± 4.87	29.9 ± 4.71	.490	.021	1.01 (1.00–1.03)	.078
HbA1C	7.1 ± 1.32	7.2 ± 1.39	.025	.074	.93 (.88–.99)	.012
Total cholesterol	5.15 ± 1.153	5.24 ± 1.358	.038	.071	N/A	N/A
LDL	2.92 ± .972	3.00 ± 1.050	.011	.079	.92 (.86–.99)	.034
HDL	1.34 ± .326	1.32 ± .323	.112	.062	1.11 (.88–1.41)	.380
Triglycerides	2.20 ± .067	2.22 ± 1.803	.806	.016	1.03 (.99–1.07)	.224

**Table 2:** Demographic and clinical variables in patients who accepted treatment and those who did not.

	Descriptives				Multivariate	
	Participating (*n* = 191)	Not participating (*n* = 91)			Participating = reference	
	Mean ± SD (%)	Mean ± SD (%)	*P*	Cohen's *d *	OR (95% CI)	*P*
Gender						
(female = reference)	(55)	(39)	.074	N/A	1.592 (.833–3.041)	.159
Age (years)	58.4 ± 5.41	57.7 ± 6.15	.310	.121	.979 (.923–1.039)	.482
Education (years)	12.4 ± 2.45	12.0 ± 2.49	.272	.162	1.205 (1.053–1.379)	.007
Professional status						
(active = reference)	(30.4)	(44.9)	.022	N/A	3.241 (1.530–6.865)	.002
Socioeconomic status						
(good = reference)	(43.3)	(36.2)				
Average	(44.9)	(47.3)	.399	N/A	.864 (.450–1.658)	.660
Poor	(11.8)	(16.5)			.787 (.311–1.991)	.613
Family status						
(married = reference)	(74.6)	(78.0)	.556	N/A	.812 (.399–1.654)	.567
Acute stress	(42.6)	(39.5)	.684	N/A	N/A	
Chronic stress						
(yes = reference)	(58.2)	(60.0)	.880	N/A	N/A	
BMI (kg/m^2^)	29.7 ± 4.29	31.03 ± 4.86	.024	.290	.910 (.850–.975)	.007
HbA1C (%)	7.1 ± 1.30	7.0 ± 1.28	.437	.078	1.246 (.972–1.597)	.082
Total cholesterol (mmol/L)	5.20 ± 1.116	5.08 ± 1.006	.417	.113	N/A	
LDL cholesterol (mmol/L)	2.97 ± .994	2.87 ± .852	.458	.108	1.112 (.803–1.539)	.522
HDL cholesterol (mmol/L)	1.40 ± .300	1.34 ± .339	.108	.187	.549 (.183–1.645)	.284
Triglycerides (mmol/L)	1.87 ± .965	2.44 ± 2.997	.023	.256	.838 (.692–1.016)	.072
